# Modelling the impact of incarceration and prison‐based hepatitis C virus (HCV) treatment on HCV transmission among people who inject drugs in Scotland

**DOI:** 10.1111/add.13783

**Published:** 2017-03-03

**Authors:** Jack Stone, Natasha K. Martin, Matthew Hickman, Sharon J. Hutchinson, Esther Aspinall, Avril Taylor, Alison Munro, Karen Dunleavy, Erica Peters, Peter Bramley, Peter C. Hayes, David J. Goldberg, Peter Vickerman

**Affiliations:** ^1^ School of Social and Community MedicineUniversity of Bristol, BristolUK; ^2^ Division of Global Public HealthUniversity of California San DiegoSan DiegoCAUSA; ^3^ School of Health and Life SciencesGlasgow Caledonian University, GlasgowUK; ^4^ Health Protection ScotlandGlasgowUK; ^5^ School of Media, Culture and SocietyUniversity of the West of Scotland, PaisleyUK; ^6^ Gartnavel General HospitalGlasgowUK; ^7^ NHS Forth Valley Viral Hepatitis Service, StirlingUK; ^8^ Division of Health SciencesRoyal Infirmary Edinburgh, EdinburghUK

**Keywords:** DAAs, HCV, mathematical model, OST, people who inject drugs, prison

## Abstract

**Background and Aims:**

People who inject drugs (PWID) experience high incarceration rates, and previous incarceration is associated with elevated hepatitis C virus (HCV) transmission risk. In Scotland, national survey data indicate lower HCV incidence in prison than the community (4.3 versus 7.3 per 100 person‐years), but a 2.3‐fold elevated transmission risk among recently released (< 6 months) PWID. We evaluated the contribution of incarceration to HCV transmission among PWID and the impact of prison‐related prevention interventions, including scaling‐up direct‐acting antivirals (DAAs) in prison.

**Design:**

Dynamic mathematical modelling of incarceration and HCV transmission, using approximate Bayesian computation for model calibration.

**Setting:**

Scotland, UK.

**Participants:**

A simulated population of PWID.

**Measurements:**

Population‐attributable fraction (PAF) of incarceration to HCV transmission among PWID. Decrease in HCV incidence and chronic prevalence due to current levels of prison opiate substitution therapy (OST; 57% coverage) and HCV treatment, as well as scaling‐up DAAs in prison and/or preventing the elevated risk associated with prison release.

**Findings:**

Incarceration contributes 27.7% [PAF; 95% credible interval (CrI) –3.1 to 51.1%] of HCV transmission among PWID in Scotland. During the next 15 years, current HCV treatment rates (10.4/6.8 per 1000 incarcerated/community PWID annually), with existing prison OST, could reduce incidence and chronic prevalence among all PWID by a relative 10.7% (95% CrI = 8.4–13.3%) and 9.7% (95% CrI = 7.7–12.1%), respectively. Conversely, without prison OST, HCV incidence and chronic prevalence would decrease by 3.1% (95% CrI = –28.5 to 18.0%) and 4.7% (95% CrI = –11.3 to 14.5%). Additionally, preventing the heightened risk among recently released PWID could reduce incidence and chronic prevalence by 45.0% (95% CrI = 19.7–57.5%) and 33.3% (95% CrI = 15.6–43.6%) or scaling‐up prison HCV treatments to 80% of chronic PWID prison entrants with sufficient sentences (>16 weeks) could reduce incidence and prevalence by 45.6% (95% CrI = 38.0–51.3%) and 45.5% (95% CrI = 39.3–51.0%), respectively.

**Conclusions:**

Incarceration and the elevated transmission risk following prison release can contribute significantly to hepatitis C virus transmission among people who inject drugs. Scaling‐up hepatitis C virus treatment in prison can provide important prevention benefits.

## Introduction

Hepatitis C virus (HCV) is a blood‐borne disease causing considerable morbidity 1. Injecting drug use is the primary mode of HCV transmission in many developed countries 2, with approximately half of people who inject drugs (PWID) infected with HCV 3. Strategies to control HCV transmission among PWID, therefore, are critical to preventing HCV in the population.

Globally, PWID experience high incarceration rates (56–90% ever being incarcerated 4), and previous incarceration is associated frequently with HCV infection 5 and increased injecting risk in the community 6, 7. Recent prison release is also associated with heightened transmission risk 8. HCV incidence among incarcerated PWID [4.3–34 per 100 person‐years (py)] 9, 10 varies greatly world‐wide.

Prison could, be an important setting to deliver HCV prevention interventions, although few countries currently do this 4, 11, 12. In Spanish prisons, PWID experience a fivefold lower incidence if on OST 13. Similarly, after introducing prison OST in Scotland, current coverage of 57% among PWID 9, evidence suggests HCV incidence among incarcerated PWID reduced 9, 14, and is now lower than among community PWID 15. HCV treatment for incarcerated PWID, especially with shorter direct‐acting antivirals (DAAs 16), could reduce HCV transmission in prison and the community. However, although modelling suggests testing and treatment with DAAs could be cost‐effective in UK prisons 17, HCV treatment in prison remains low 11.

In this study, we evaluate the importance of prison as a setting to undertake HCV prevention interventions for PWID in Scotland. Specifically, we aim to:

1. Estimate the contribution of incarceration to the Scottish HCV epidemic among PWID.
2. Estimate the 15‐year impact of existing prison‐based prevention and HCV treatment interventions on HCV incidence and chronic prevalence among PWID.
3. Estimate the 15‐year impact of potential future prison‐associated prevention and HCV treatment interventions on HCV incidence and chronic prevalence.

## Methods

### Setting

This study took place in Scotland, where 61% of PWID have ever been incarcerated with an average sentence length of 5.6 months, and HCV incidence among PWID is lower in prison than community, but PWID released in the last 6 months have a greater risk of HCV acquisition than other community PWID.

### Design

To address these aims, we developed a mathematical model of HCV transmission and incarceration among PWID (see Model description below) which, where possible, was fitted to detailed data from Scotland. Model parameterization and calibration comprised two stages. In stage 1, incarceration dynamics were parameterized and calibrated to self‐reported data from community PWID on their incarceration history using Bayesian methodology that incorporated uncertainty in both the inputs and the outputs (approximate Bayesian computation sequential Monte Carlo scheme 23). In stage 2, the HCV transmission component was parameterized, utilizing results from the relevant literature, and calibrated to recent data on the HCV incidence and prevalence among PWID in Scotland (see Model parameterization and calibration for further details).

The model was used to estimate the contribution of incarceration to the Scottish HCV epidemic among PWID, the ‘population‐attributable fraction’ (PAF). The World Health Organization defines PAF as the ‘proportional reduction in population disease or mortality that would occur if exposure to a risk factor were reduced to an alternative ideal exposure scenario’ 18. We estimate this PAF by considering the relative reduction in endemic HCV incidence if there were no differences in HCV transmission risk during incarceration or post‐release; by considering endemic HCV incidence, our estimate incorporates the impact that these differences in risk have on elevating the whole epidemic. The model also estimates the 15‐year impact on HCV incidence and prevalence of existing prison‐based interventions: HCV treatment and OST. The model is used to project the impact of potential future prison‐associated prevention and HCV treatment interventions: preventing future incarceration of PWID, the scaling‐up of HCV treatment on entry into prison and/or the prevention of the elevated risk following prison release (see Model analyses for further details).

### Model description

**Figure 1:**
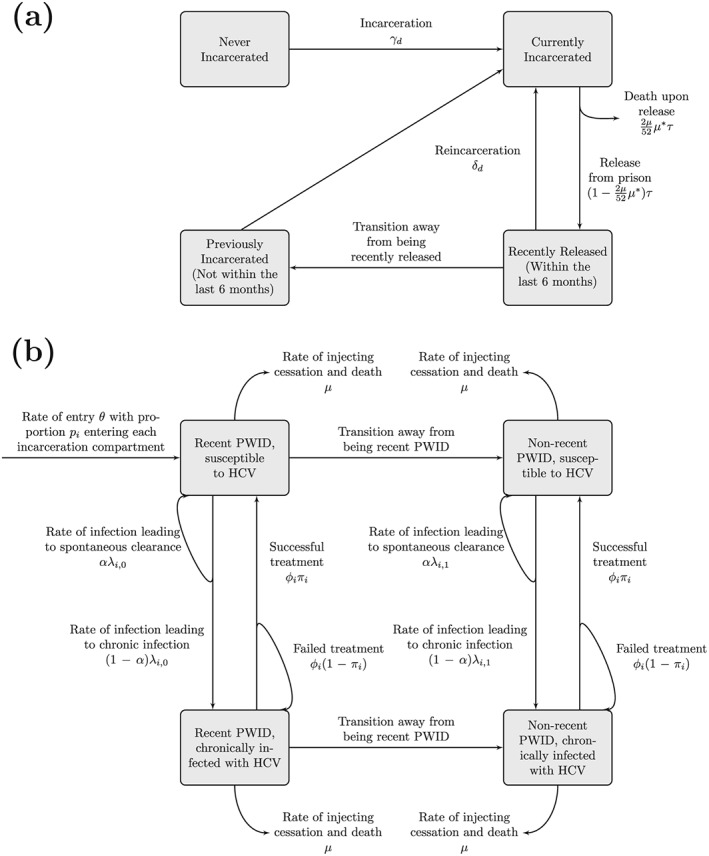
Schematic of model components for (a) people who inject drugs (PWID) incarceration and (b) hepatitis C virus (HCV) transmission

We developed a dynamic, deterministic model of incarceration and HCV transmission among current PWID (schematic in Fig. 1; model equations in section 1 of the Supporting information). The PWID population was stratified by incarceration state (never, currently, recently and non‐recently released from prison; within the last 6 months or not, respectively), HCV infection state (susceptible and chronically infected) and injecting duration (recent (< 5 years) and non‐recent (> 5 years) injectors). The model is open; PWID enter through drug use initiation, and leave either through permanent cessation of injecting or death, with excess mortality following prison release 19.

PWID are incarcerated or reincarcerated at rates which vary by duration of injecting, and are released from prison at a constant rate. A small proportion of those released from prison leave the model due to mortality on release, while the remainder enter the recently released compartment where they experience elevated HCV acquisition risk for 6 months before transitioning to the non‐recent previously incarcerated compartment.

All PWID can acquire and transmit HCV in their given setting (prison or community). Susceptible PWID become infected at a rate proportional to the chronic prevalence in their setting and the infection rate. The infection rate varies by setting, by whether or not a PWID has recently initiated injecting, and is elevated if a PWID has been recently released from prison. A proportion of those acutely infected clear infection spontaneously and remain in the susceptible compartment, while the remainder proceed to chronic infection. The model does not include a compartment for acutely infected PWID because previous modelling indicates that it contributes little to transmission 20, 21.

A fixed number of chronically infected PWID are treated in the community and prison annually. If prison HCV treatment rates exceed the number of eligible chronically infected incarcerated PWID, defined to be those infected PWID with long enough sentences to complete treatment, then all eligible PWID are treated. A proportion of treated PWID achieve sustained viral response (SVR) and become susceptible, while those failing treatment remain chronically infected. The SVR rates are time‐dependent and setting‐specific. We model HCV treatment as instantaneous, because of the short duration of DAA treatment regimens 16. PWID failing treatment are eligible for retreatment due to the wide range of HCV treatment options becoming available 16.

### Model parameterization and calibration

Where possible, the model was fitted to detailed data from Scotland. Data for parameterizing and calibrating the models came either from a national cross‐sectional sero‐behavioural survey of Scotland's closed prisons (2010/11, denoted as the ‘prison survey’) 9, or the Needle Exchange Surveillance Initiative (NESI), a series of four cross‐sectional surveys of community PWID in Scotland between 2008 and 2014 8, 22.

#### Stage 1: Parameterizing and calibrating the incarceration submodel

We tracked a simulated cohort of 1000 PWID for 20 years from initiation of injecting to calibrate the model's incarceration and reincarceration rates, and proportion of new PWID initiating injecting in each incarceration state. An approximate Bayesian computation sequential Monte Carlo scheme 23 was used to obtain a sample of 10 000 incarceration‐related parameter sets (prior distributions and posterior parameter ranges in Table 1) that sufficiently fitted the NESI incarceration data on the proportions of community PWID who have never been incarcerated, incarcerated once or multiple times by duration injecting (data used shown in Supporting information, Table S1), while also giving a total PWID population size within the latest estimates 26. Full details of this calibration process are in section 2 of the Supporting information.

**Table 1:** Posterior model parameter ranges used in the full model, obtained through the incarceration submodel calibration.

Parameter	Symbol	Prior distribution	Posterior parameter range	Source and comments
Death rate (per year)^a^	μ_1_	Sampled from a Poisson distribution with mean (10), with sampled values divided by 1000	0.006–0.014	24
Average duration injecting (years)^a^	μ_2_	Uniform on (5,20)	5.1–17.7	25
Factor increase in mortality rate for 2 weeks following prison release	μ*	Log‐normal with parameters (2.0053, 0.1393) truncated to 95% CI = 5.7, 9.9	6.3–8.2	19
Percentage of prison population that are current PWID	P	Normal with parameters (0.19, 0.006) truncated to 95% CI = 0.18–0.21	18.7–21.0%	Scottish prison survey 9
Current PWID population size	*n*	NA	15286–18600	Parameter sets are rejected if model population size not within 11 500–18 600 26
Percentage of PWID initiating injecting when^b^		Dirichlet distribution with parameters (10,1,1,1,1)		Obtained through model fitting
Never incarcerated	p_1_		72.2–92.5%	
Incarcerated for first time	p_2_		1.6–12.0%	
Community, incarcerated once	p_3_		1.4–10.3%	
Incarcerated for second or more time	p_4_		3.2–13.7%	
Community, incarcerated twice or more	p_5_		0.2–8.3%	
Incarceration rates per year	γ			Obtained through model fitting
Recent PWID (< 5 years injecting)		Uniform on (0, 0.25)	0.12–0.17	
Non‐recent PWID (> 5 years injecting)		Uniform on (0, 0.25)	0.03–0.06	
Re‐incarceration rates per year	δ			Obtained through model fitting
Recent PWID		Uniform on (0,1)	0.63–0.88	
Non‐recent PWID		Uniform on (0,1)	0.08–0.17	
Release rate per year	τ	Normal with parameters (0.48, 0.019) truncated to 95% CI = 0.44–0.52	0.47–0.51	Scottish prison survey^c^ Corresponds to an average 5.7–6.1 months spent in prison per incarceration

a The PWID leaving rate, μ, is given by: μ_1_ + 1/ μ_2_.

b In the final model which does not stratify incarceration history into incarcerated once and twice or more, p_2_ and p_4_ are combined to give the proportion of people who inject drugs (PWID) initiating injecting in prison, while p_3_ and p_5_ are combined to give the proportion of PWID initiating injecting in the community having been incarcerated ‐ a random proportion of which have been released recently.

c We used the weighted average time between date of incarceration and earliest date of liberty for current PWID, i.e*.* weighted by the reciprocal of these times to allow for the probable oversampling of prisoners with long sentences. CI = confidence interval; NA = not applicable.

#### Stage 2: Parameterizing and calibrating the full model

Parameters for the full model are shown in Tables 1 and 2. For each of the 10 000 parameter fits for the incarceration submodel, the HCV transmission component of the full model was calibrated to sampled HCV incidences (from distribution ranges given in Table 2) for recent and non‐recent community PWID (2008) and incarcerated PWID (2010) (more details in section 3 of the Supporting information). Parameter sets were accepted as model fits if the resulting model projections for the HCV prevalence among community PWID and incarcerated PWID lay within the 95% confidence intervals (CI) of the corresponding data for NESI 2008 and the prison survey (2010/11), respectively. We assumed that the HCV epidemic was stable (in steady‐state) prior to the scale‐up of HCV treatment in 2008. The model assumed a factor increase in community HCV acquisition risk among PWID recently released (< 6 months) from prison (2.30, 95% CI = 0.97–5.46; details in section 3.1 of the Supporting information).

**Table 2:** Full model parameters obtained from literature and data analyses**.**

Parameter	Symbol	Range of parameter values	Source and comments
Inflow of new injectors per year	*θ*	–	Fitted to PWID population size
HCV incidence among PWID per 100 person‐years (2008 unless stated otherwise)	Vary infection rate, λ, to fit		Estimated from NESI data 22 and prison survey 9. See section 3.2 of the Supporting information. HCV incidences are sampled from the distributions obtained by a bootstrapping method to estimate the 95 CIs Incidence among incarcerated PWID in absence of OST is sampled from log‐normal (2.3,0.6) which is truncated to the 95% CI (4.5,31.8) found in a previous prison survey before OST was introduced 14.
Recent community PWID (< 5 years injecting)		11.9–40.6	
Non‐recent community PWID (> 5 years injecting)		4.8–19.5	
Incarcerated PWID with OST (2010/11)		0.9–10.2	
Incarcerated PWID without OST		4.5–31.8	
HCV antibody prevalence			
Community PWID (2008)		49.7–54.0%	22
Incarcerated PWID (2010/11)		51.0–55.9%	9
Proportion of new infections that spontaneously clear	α	0.22–0.29	27 Sampled from uniform distribution
Annual PWID treatments in community (average rate per 1000 community PWID)	Φ_c_		28, 29
2008–14		66–103 (4.4–6.8)	
2015–30		103 (6.8)	
Annual PWID treatments in prison (average rate per 1000 incarcerated PWID)	Φ_p_		28, 29
2008–14		4–16 (2.6–10.4)	
2015–2030		Varied	
Sustained viral response	π		
PEG‐IFN/RBV in community		60–66%	30 Sampled from uniform distribution
PEG‐IFN/RBV in prison		55–66%	30 Sampled from uniform distribution
DAAs (2015–30)		90%	16
Percentage of incarcerated PWID with sentences:			
> 16 weeks	ε	39.9–46.0%	Estimated from the prison survey. Both sampled from normal distribution
> 12 weeks	ε_2_	57.3–63.3%	
Increased risk among recently released PWID (< 6 months since release)	η	0.97–5.46	Estimated from NESI data (see section 3.1 of the Supporting information). Sampled from log‐normal distribution

CI = confidence interval; PWID = people who inject drugs; OST = opiate substitution therapy; NESI = Needle Exchange Surveillance Initiative; PEG‐IFN/RBV = pegylated interferon and ribavirin; DAA = direct‐acting antiviral.

Annual rates of HCV treatment for incarcerated and community PWID in Scotland were estimated for 2008–14 from their national treatment database 28, 29, 30. Community and prison SVR rates for HCV treatment with pegylated interferon (PEG‐IFN) and ribavirin during 2008–14 were parameterized based on recent analyses of HCV treatment outcomes among Scottish patients 30, which found lower (albeit not significantly lower) SVR rates among incarcerated patients initiating treatment. From 2015, we assume HCV treatment with DAAs and assume only those with sufficiently long sentences are treated, with no difference in the SVR rates between prison and community.

### Model analyses

#### Contribution of incarceration to the Scottish HCV epidemic among PWID

Using the calibrated model, we projected the contribution or PAF of incarceration to current HCV transmission among PWID in Scotland. We compared the endemic HCV incidence in the baseline epidemic (reduced HCV transmission in prison compared to the community, but elevated HCV acquisition risk in the 6‐month period post‐release) with the projected HCV incidence resulting from a scenario where there is no effect of incarceration on HCV transmission risk. This was modelled by increasing the prison HCV transmission risk to the same as the community and assuming no excess risk among recently released PWID. In both scenarios, the model was run to the stable endemic state, with the relative difference between the endemic HCV incidence for the ‘no effect of incarceration’ scenario and the baseline scenario being defined as the PAF of incarceration to HCV transmission.

#### Impact of existing prevention and HCV treatment interventions

First, we projected the *status quo* HCV epidemic among PWID until 2030, including existing interventions (current levels of in‐prison and community HCV treatment, with IFN‐free DAAs being used from the start of 2015, and lower incidence in prison compared to the community). Secondly, we projected how the impact would change if in‐prison HCV treatment was ceased from 2015. Thirdly, we projected the impact of the existing OST programme in Scottish prisons, by assuming an HCV incidence among incarcerated PWID observed prior to initiation of prison OST in a long‐stay prison in Scotland from 2015 onwards.

#### Impact of potential future prison‐associated prevention and HCV treatment interventions

We additionally projected the impact of future prevention and HCV treatment interventions. First, we evaluated the potential impact of decriminalization by considering a theoretical scenario where there are no new incarcerations of PWID from 2015. We modelled this by turning off incarceration and reincarceration in the model (i.e*.* set the rates to 0) but with people still initiating injecting in prison, while HCV treatment of community PWID continued at the same rate per 1000 PWID as in the *status quo* scenario, i.e. an increased number of annual treatments. Secondly, we simulated a potential new intervention strategy that prevents the elevated transmission risk post‐release, estimated by comparing the *status quo* epidemic with a scenario where there is no elevated risk post‐release. Then, we projected the impact of scaling‐up in‐prison HCV treatment from 2015. Specifically, the following scenarios were modelled:

1. Immediate scale‐up of HCV treatment to 80% of chronically infected PWID with at least 16‐week sentences treated immediately on prison entry (43% of imprisoned PWID). A 16‐week sentence was assumed to be the minimum time needed to diagnose, assess and treat someone with a 12‐week DAA treatment course.
2. Immediate scale‐up of HCV treatment to 80% of chronically infected PWID with at least 12‐week sentences treated immediately on prison entry (60% of imprisoned PWID). This assumes an 8‐week DAA treatment course.

We projected the impact of these scaled‐up HCV treatment scenarios with and without the immediate prevention of the elevated transmission risk post‐release.

### Uncertainty analysis

We undertook a linear regression analysis of covariance to determine which parameter uncertainties contribute most to uncertainty in the 15‐year impact of scaling‐up annual prison HCV treatment rates so that 80% of chronically infected PWID with at least 16‐week sentences are treated on prison entry from 2015. The proportion of each model outcome's sum‐of‐squares contributed by each parameter was calculated to estimate the importance of individual parameters to the overall uncertainty.

## Results

### Contribution of incarceration to the Scottish HCV epidemic

**Figure 2:**
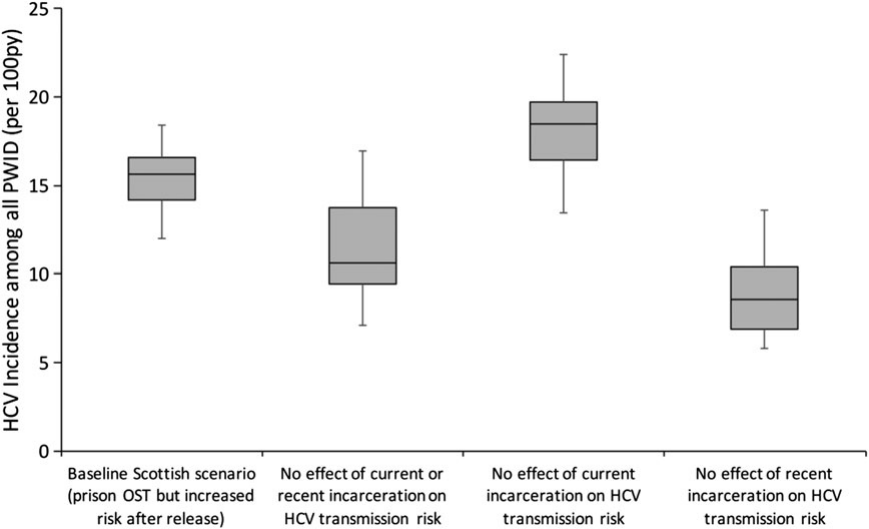
Endemic hepatitis C virus (HCV) incidence among all people who inject drugs (PWID) with various effects of incarceration removed. Boxes indicate the interquartile range, with the lines inside indicating the median incidence, with whiskers representing 95% credible interval (CrI) for the simulations. OST = opioid substitution therapy

Our baseline projections for Scotland predicted an overall HCV incidence among PWID of 15.6 per 100 py [95% credible interval (CrI) = 12.0–18.4] in 2008 (Fig. 2). HCV incidence would be 27.7% (95% CrI = –3.1 to 51.1%) lower at 10.6 per 100 py (95% CrI = 7.1–17.0) if incarceration had no effect on HCV transmission. Hence, despite lower HCV incidence in prison than the community, incarceration of PWID contributes nearly a third of all current HCV transmission among PWID (i.e. the PAF of incarceration is 27.7%). This is due purely to the heightened risk post‐release, as shown in Fig. 2.

### Impact of existing prevention and HCV treatment interventions

**Figure 3:**
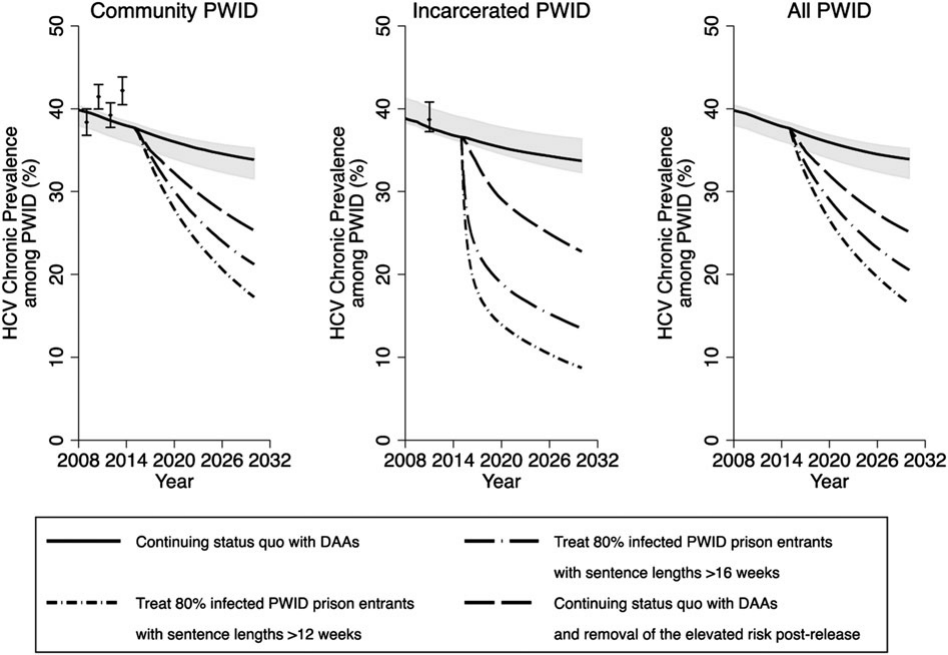
Impact of different prevention and treatment scenarios on chronic hepatitis C virus (HCV) prevalence over time in Scotland among community people who inject drugs (PWID), incarcerated PWID and all PWID. Lines represent the median chronic HCV prevalence, with the shaded area representing the 95% credible interval (CrI) for the *status quo* projection (no scale‐up) from 2015 onwards. HCV prevalence data points shown for comparison with 95% confidence intervals. DAA = direct‐acting antiviral

**Figure 4:**
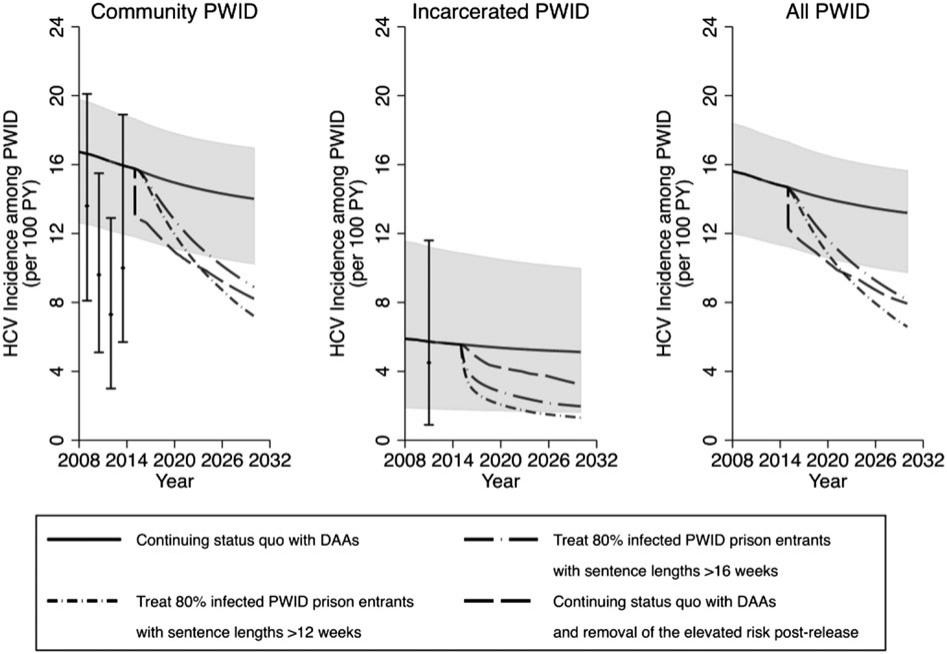
Impact of different prevention and treatment scenarios on hepatitis C virus (HCV) incidence over time in Scotland among community people who inject drugs (PWID), incarcerated PWID and all PWID. Lines represent the median HCV incidence, with the shaded area representing the 95% credible interval (CrI) for the *status quo* projection (no scale‐up) from 2015 onwards. HCV incidence data points shown for comparison with 95% confidence intervals. DAA = direct‐acting antiviral

**Figure 5:**
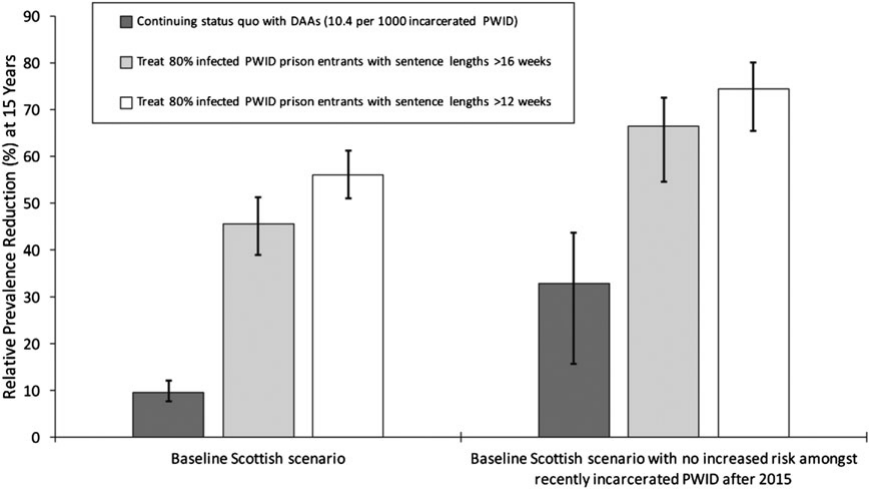
Relative chronic prevalence reduction among all people who inject drugs (PWID) from 2015 to 2030 for different prison treatment scenarios, with or without the concurrent removal (from 2015) of the heightened hepatitis C virus (HCV) transmission risk among recently released PWID. Bars indicate median chronic prevalence reduction, with whiskers representing the 95% credible interval (CrI) for the projections. DAA = direct‐acting antiviral

**Figure 6:**
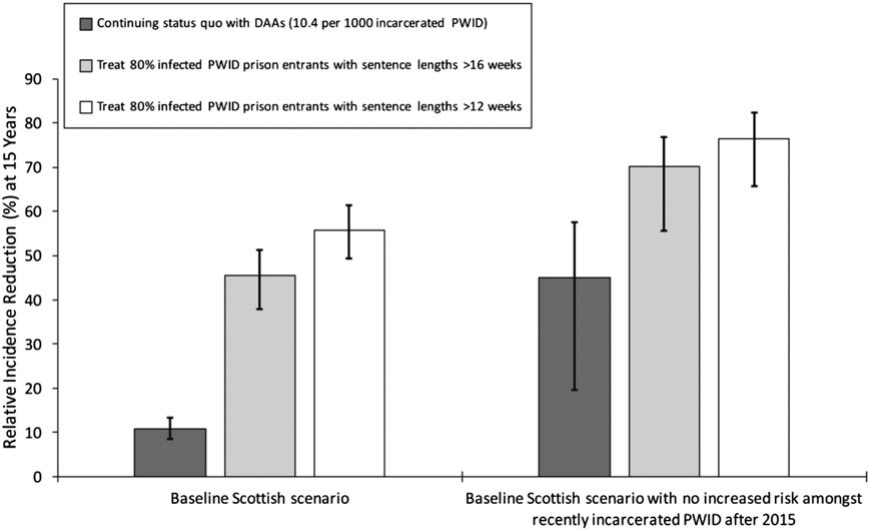
Relative incidence reduction among all people who inject drugs (PWID) from 2015 to 2030 for different prison treatment scenarios, with or without the concurrent removal (from 2015) of the heightened hepatitis C virus (HCV) transmission risk among recently released PWID. Bars indicate median incidence reduction, with whiskers representing the 95% credible interval (CrI) for the projections. DAA = direct‐acting antiviral

In the *status quo* scenario, maintaining current HCV treatment rates (average annual rates of 10.4 and 6.8 per 1000 incarcerated and community PWID, respectively) with DAAs decreases overall HCV incidence and chronic prevalence among PWID over 15 years by a relative 10.7% (95% CrI = 8.4–13.3%) and 9.7% (95% CrI = 7.7–12.1%), respectively (Figs 3, 4, 5, 6), with prevalence decreasing from 37.6% (95% CrI = 35.8–38.3%) in 2015 to 33.9% (95% CrI = 31.6–35.2%) in 2030. Conversely, if no prison HCV treatment occurred from 2015 onwards, then incidence and chronic prevalence would still decrease due to continued community DAA treatment, by a relative 10.0% (95% CrI = 8.4–11.6%) and 8.9% (95% CrI = 7.2–11.2%), respectively, over 15 years. Additionally, without current coverage levels of prison OST, HCV incidence and chronic prevalence would still decrease, but by only a relative 3.1% (95% CrI = –28.5 to 18.0%) and 4.7% (95% CrI = –11.3 to 14.5%), respectively, over 15 years, with incidence being 9.3% (95% CrI = –7.2 to 46.6%) higher than the *status quo* scenario in 2030.

### Impact of potential future prison‐associated prevention and HCV treatment interventions

Preventing future incarceration of PWID from 2015, along with current HCV treatment rates, could reduce HCV incidence and chronic prevalence by 21.9% (95% CrI = 4.8–38.5%) and 16.9% (95% CrI = 6.1–27.9%), respectively, by 2030. Conversely, pairing current HCV treatment and prison OST with an intervention that prevented the heightened risk among recently released PWID could decrease incidence and chronic prevalence further by 45.0% (95% CrI = 19.7–57.5%) and 33.3% (95% CrI = 15.6–43.6%), respectively (Figs 5 and 6), over 15 years. Alternatively, if prison treatment rates are scaled‐up, so that 80% of all chronically infected PWID with sentences longer than 16 weeks are treated on prison entry, then HCV incidence and chronic prevalence (Figs 5 and 6) would reduce by 45.6% (95% CrI = 38.0–51.3%) and 45.5% (95% CrI = 39.3–51.0%), respectively, by 2030. If the heightened transmission risk among recently released PWID is also prevented, then incidence and chronic prevalence could reduce further by up to 70.2% (95% CrI = 55.0–77.4%) and 66.5% (95% CrI = 51.4–70.1%), respectively. Conversely, if 80% of chronically infected PWID entering prison with sentences longer than 12 weeks could be treated, then HCV incidence and chronic prevalence would reduce by 55.8% (95% CrI = 49.3–61.4%) and 55.9% (95% CrI = 51.1–61.3%), respectively, over 15 years if there was no reduction in the HCV risk post‐release, or 76.4% (95% CrI = 65.6–82.2%) and 74.4% (95% CrI = 61.8–77.3%) if this risk was also prevented.

### Uncertainty analysis

Analysis of covariance indicated that uncertainty in the heightened risk among recently released PWID (accounts for 17 and 12% of uncertainty, respectively), HCV transmission rate among non‐recent community PWID (24 and 25%) and the proportion of incarcerated PWID eligible for HCV treatment (18 and 20%) contributed most to the uncertainty in the impact of scaling‐up prison HCV treatment rates on overall PWID HCV incidence and chronic prevalence from 2015 to 2030. No other model parameters contributed more than 10% to the uncertainty (see section 4 of the Supporting information).

## Discussion

### Main findings

Model projections suggest that, despite lower HCV transmission risk during imprisonment than the community, nearly a third of current HCV transmission among Scottish PWID could be attributed to incarceration. This is due primarily to the elevated HCV risk post‐release, with the model suggesting that HCV incidence could be reduced by 45% over the next 15 years if this risk was prevented. Less impact would be achieved by preventing future incarcerations of PWID (e.g. by decriminalization), a 22% reduction in HCV incidence over 15 years, due to current incarceration being associated with low HCV transmission risk. Conversely, continuing with current levels of HCV treatment among PWID over the next 15 years will have only a modest prevention impact, reducing HCV incidence and chronic prevalence by approximately one‐tenth. In contrast, if 80% of infected prisoners with sentences longer than 16 weeks were treated, HCV incidence and chronic prevalence among PWID in Scotland could be almost halved in 15 years. If this scale‐up in HCV treatment could also be combined with an intervention preventing the elevated HCV risk post‐release, prevalence and incidence could reduce further by nearly three‐quarters to 13.6% and 4.2 per 100 py, respectively.

### Limitations

Our study had several limitations. Firstly, our findings may not be directly generalizable to other settings, as our model was parameterized to Scotland. Nonetheless Scotland is one of few sites with detailed national data on community and prison HCV incidence and prevalence, as well as detailed incarceration data that enable such a detailed evaluation of the role of incarceration. In settings with higher HCV incidence in prison, greater incarceration rates and longer sentences than Scotland (61% of PWID have ever been incarcerated with average sentence length of 5.6 months), e.g. Thailand, incarceration is likely to contribute more to HCV transmission 31.

Secondly, our model projections assumed stable levels of HCV treatment among community PWID for 2015–30 29. Although community treatment rates may increase in coming years with the greater availability of DAAs, which would achieve greater impact on HCV transmission, we did not consider this because it was not the focus of our study.

Thirdly, our analyses suggest the elevated HCV risk post‐release may be an important contributor to the current HCV epidemic among PWID in Scotland, but uncertainty exists over the magnitude and duration of this risk. However, although the odds ratio for the elevated HCV acquisition risk post‐release is not statistically significant (*P* = 0.059), other statistical analyses based on the same data set provide a consistent picture, with recent incarceration being associated with greater injecting risk (injecting daily and sharing needles or syringes in the last 6 months—unpublished analyses) and lower coverage levels of OST and needle and syringe programmes (NSP). Furthermore, the model's posterior range for elevated transmission risk post‐release is 1.17–5.24, suggesting that the model agrees with observed prevalence data only when there is an increased risk following release. It is also uncertain whether some of the heightened risk associated with recent release may occur during the period of incarceration. However, this is unlikely considering the low HCV incidence observed in Scottish prisons 9. Although other studies have observed similar heightened risks or behaviours among PWID recently released from prison 6, 7, it is important that further research determines more clearly the magnitude and reasons for this heightened risk. Additionally, we model optimistic intervention scenarios where the elevated HCV risk post‐release is fully prevented to show the potential benefit of prevention interventions targeting this important period of risk. Although studies have shown that OST and NSP are highly effective at reducing an individual's risk of acquiring HCV (in combination, up to 80% 32, 33), it is unclear whether all the elevated risk post‐release could be prevented, even with intensive prevention efforts upon prison release. Indeed, it is likely that other structural factors may also need to be addressed, including high levels of homelessness following release 8, 22, 34, to fully prevent this period of elevated risk. However, our results suggest that efforts to reduce this risk, which may include linking PWID to harm reduction services and providing housing support on release from prison, could greatly reduce both HCV incidence and prevalence.

Fourthly, our estimates of the impact of ongoing in‐prison OST for reducing HCV transmission may be underestimated if HCV transmission risk without this intervention was higher than the historical estimate from a long‐stay Scottish prison (11.9 per 100 py in 1999/2000 14) used in our counterfactual scenario. As reported in Australia 35, it is possible that individuals with shorter incarceration durations may have greater acquisition risk. Although our projections suggest that existing prison OST may be having little impact on the overall epidemic due to the low proportion (9%) of PWID in prison at any point in time, it is still likely to be cost‐effective because of the large reduction in HCV incidence and other benefits achieved (e.g. reduction in drug‐related deaths 36). Importantly, prison OST is likely to have a greater impact in other settings, where PWID experience greater rates of incarceration and longer sentences 31, e.g. Ukraine.

Lastly, we explore the possible impact of decriminalization by considering a scenario in which there are no new incarcerations of PWID. Although it is unlikely that all incarcerations of PWID would be prevented by decriminalization alone, this scenario is used to demonstrate the potential impact that decriminalization could have in reducing HCV transmission among PWID.

### Comparisons with existing studies

The work is consistent with previous modelling considering the impact of OST and HCV treatment as prevention among community PWID 21, 37, 38, and with models of the cost‐effectiveness of HCV case‐finding in prison 17, 39. Furthermore, our work is consistent with recent modelling which evaluated the impact of scaling‐up HCV treatment in US prisons 40. However, in contrast to the US study, our model is based upon detailed empirical data on differences in transmission risk in community and prison, including increased risk post‐release, as well as detailed data on the incarceration dynamics of PWID. Our study is the first to consider the implications of HCV transmission risk being elevated post‐release, and the potential impact of interventions that prevent this risk. A review found that the high cost of DAAs is a key barrier in scaling‐up HCV treatment for prevention in PWID and prisoners, while short prison sentences for PWID in many settings may have limited the prevention impact of HCV treatment in prisons 41. Our study indicates that in the DAA era, a substantial proportion of PWID prisoners in Scotland (> 40%) have sufficiently long sentences for completing treatment (16 weeks), supporting the hypothesis that prison‐based HCV treatment could now be highly effective and cost‐effective.

### Implications

It is recognized widely that the period immediately after prison poses an increased risk for drug‐related deaths 19. Our findings raise the hypothesis that this is also a critical period of HCV transmission, contributing substantially to HCV risk in the community. Further research and syntheses of available evidence are required to better define this risk. The reasons for the increased risk post‐release are likely to be multi‐factorial, associated with injecting risk environment and individual behaviours; for example, relapse may be unplanned and not involve sterile equipment, and post‐release PWID may be more likely to have unstable housing 8, 22, 34 or be unemployed 6. Additionally, they may experience changes in social networks and inadequate family and financial support 42, 43. This highlights further the detrimental effects associated with incarceration and the high societal costs of drug prohibition 31. Our findings also suggest that reduced incarceration among PWID is likely to reduce HCV prevalence and incidence. Policy changes that would reduce incarceration are also likely to generate cost savings to the criminal justice system (UK estimates of the life‐time crime costs per person who uses drugs were £445 000 in 2009 44) which could possibly be used to finance further treatment of community PWID, further decreasing HCV prevalence and transmission.

There is emerging evidence that leaving prison on OST can increase OST uptake in the community 45, and in combination with community OST can reduce the risk of drug‐related mortality 36. In addition, PWID in some prison settings are given naloxone upon release to reduce mortality risk 46 and sterile injecting equipment to reduce injecting risk 47. We show that it is important to determine whether these interventions can reduce HCV risk, with our modelling suggesting that the scale‐up of prison interventions could be an important part of comprehensive harm reduction programmes.

### Declaration of interests

J.S. has received a conference attendance sponsorship from Gilead. N.K.M. has received research grants from Gilead, and honoraria from Merck, AbbVie and Gilead. D.J.G. has received honoraria for educational contributions (e.g. lectures, reports) and for providing advice on aspects of hepatitis C and public health from AbbVie, Merck, Gilead, BMS and Janssen. P.V. has received research grants from Gilead. M.H. has received honoraria from Merck, Gilead and Janssen and is a co‐investigator on research grants from Gilead. S.J.H. has received honoraria from AbbVie and Gilead. A.M. has received honoraria from Merck. P.C.H. has received funding from Gilead, Roche, MSD, Abbvie, BMS and Jannsen.

## Supporting information


**Figure S1** Schematic for the incarceration submodel.
**Figure S2** Example of ABC SMC fit, along with the data points used in the fitting procedure with their 95% confidence intervals.
**Table S1** Data on the proportions of people who inject drugs (PWID) with zero, one or multiple incarcerations by duration of injecting, used in the ABC SMC.
**Table S2** Prior distributions and perturbation kernels for the ABC SMC algorithm used in the uncertainty analysis.
**Table S3** Derived hepatitis C virus (HCV) incidence among recent and non‐recent current community people who inject drugs (PWID) and incarcerated PWID.
**Table S4** Contribution of parameters to uncertainty in model projections.
