# Supplementary material for: Modelling the impact of incarceration and prison‐based hepatitis C virus (HCV) treatment on HCV transmission among people who inject drugs in Scotland
Source: Addiction. 2017 Mar 3;112(7):1302–14. doi: 10.1111/add.13783 (PMC5461206; doi:10.1111/add.13783)
Supplement: Supplementary file 1 — Figure S1 Schematic for the incarceration submodel. Figure S2 Example of ABC SMC fit, along with the data points used in the fitting procedure with their 95% confidence intervals. Table S1 Data on the proportions of people who inject drugs (PWID) with zero, one or multiple incarcerations by duration of injecting, used in the ABC SMC. Table S2 Prior distributions and perturbation kernels for the ABC SMC algorithm used in the uncertainty analysis. Table S3 Derived hepatitis C virus (HCV) incidence among recent and non‐recent current community people who inject drugs (PWID) and incarcerated PWID. Table S4 Contribution of parameters to uncertainty in model projections. [file ADD-112-1302-s001.doc]

**Modelling the impact of incarceration and prison treatment on HCV transmission among people who inject drugs in Scotland**

**Authors**: Jack Stone, Natasha K Martin, Matthew Hickman, Sharon J Hutchinson, Esther Aspinall, Avril Taylor^5^, Alison Munro, Karen Dunleavy, Erica Peters, Peter Bramley, Peter C Hayes, David J Goldberg, Peter Vickerman

**Contents**

[1 Model Equations 2](#__RefHeading___Toc465682974)

[2 Parameterising the incarceration component of the model 7](#__RefHeading___Toc465682975)

[2.1 The ABC SMC Algorithm 11](#__RefHeading___Toc465682976)

[2.2 ABC SMC to sample incarceration parameters 13](#__RefHeading___Toc465682977)

[3 Parameterising the transmission component of the model 17](#__RefHeading___Toc465682978)

[3.1 Increased Risk amongst recently incarcerated PWID 18](#__RefHeading___Toc465682979)

[3.2 HCV incidence amongst recent and non-recent PWID 19](#__RefHeading___Toc465682980)

[4 Uncertainty in Model Projections 20](#__RefHeading___Toc465682981)

[**References** 23](#__RefHeading___Toc465682982)

# Model Equations

The model includes compartments for susceptible PWID
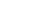
 and chronically infected PWID
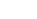
. The model population was stratified by incarceration status: never incarcerated, currently incarcerated, recently released (within the last 6 months) and previously incarcerated but not in last 6 months (i=0, 1, 2, 3 respectively). The model population was further stratified by duration of injecting: recent and non-recent PWID, <5 years and > 5 years injecting respectively (d=0 or 1 respectively).

New PWID enter the model at rate
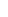
 as uninfected, recent PWID with a fixed proportion p_i_ of entrants entering into each incarceration state. PWID transition from recent to non-recent PWID with rate 0.2 per year and leave all compartments through death or permanent cessation of injecting with rate
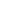
.

PWID in the community are (re)incarcerated with rates dependent upon duration of injecting and incarceration state, with never incarcerated PWID experiencing incarceration with rate
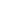
 and recently released and previously incarcerated PWID experiencing a re-incarceration rate
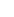
. Incarcerated PWID are released from prison with rate
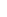
, with a proportion
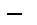
; representing a 2-week period of elevated risk of drug-related deaths post release, since this elevated risk diminishes 3-4 weeks after release) of these removed from the model to simulate increased mortality amongst drug users in the short time after their release from prison with the reminder entering the recently released compartment. PWID spend an average of 6 months in the recently released compartment before transitioning to the previously incarcerated compartment.

All PWID are initially susceptible
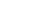
 and become HCV infected with a per-capita rate, λ_s,d_, specific to the duration of injecting and incarceration status (in community or prison, denoted by s=0 or 1 respectively). Forces of infection are given by
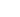
 for incarcerated PWID (constant across all duration of injecting) and
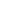
 for community PWID, specific to duration of injecting (d=0 for recent PWID and d=1 for non-recent PWID). The force of infection amongst recently released PWID is heightened by a factor
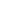
. Incarcerated PWID can only transmit HCV to other incarcerated PWID and similarly community PWID can only transmit HCV to other PWID in the community.

A proportion (
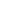
) of PWID spontaneously clear their acute infection and remain susceptible
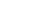
, with the remaining proportion
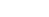
 proceeding to the chronically infected compartment
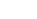
.
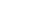
 and
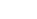
 chronically infected PWID are treated in the community and prison respectively, with a proportion
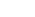
 immediately achieving SVR and transitioning to the susceptible compartment
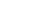
, whilst the remainder
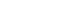
 fail treatment and remain chronically infected. If the number of PWID to be treated in the community exceed the number of chronically infected PWID than all are treated, whilst if the number to be treated exceed the number of eligible (42% corresponding to the proportion with sentence lengths of 16 weeks or greater), chronically infected, incarcerated PWID, then all eligible PWIDs are treated. Community and prison treatment rates in the model equations are given by
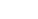
) and
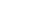
 respectively. No immunity is assumed because data are uncertain(1) and previous analyses suggested it is likely to have little effect on model projections(2, 3).

We model HCV treatment as instantaneous, because of the short duration of DAA treatment regimens(4). Although the average sentence length for PWID (5.8 months) was shorter than the duration of HCV treatment prior to 2015 (24-48 weeks), treatment rates for peg-interferon and ribavirin in prison were very low; at most 16 PWID were treated a year since the national scale-up in 2008. We use observed SVR rates amongst prisoners treated during this time which accounts for any loss to follow up due to individuals leaving prison before finishing treatment, which was not great because treatment was generally only given to those with sufficient sentence lengths to complete treatment(5).

The full model equations are as follows, for PWID who have never been incarcerated:


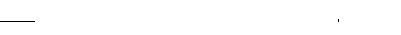


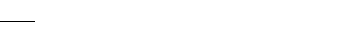


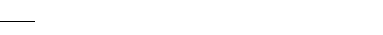


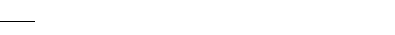


For incarcerated PWID:


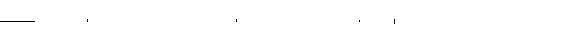


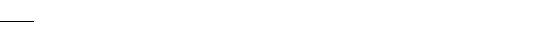


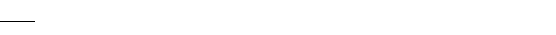


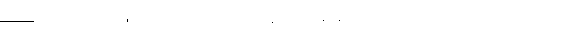


For recently incarcerated PWID:


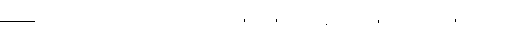


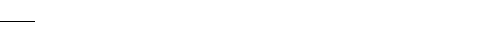


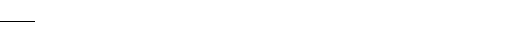


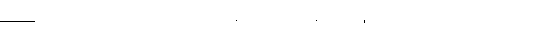


For previously incarcerated PWID:


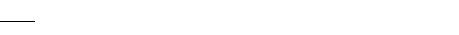


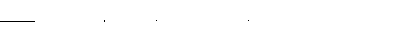


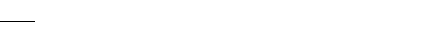


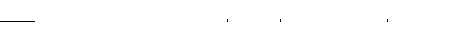


With the force of infection:


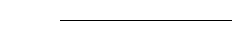


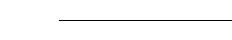


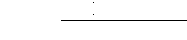


Treatments in community are allocated proportionately to population size so that:

For i=0,2,3


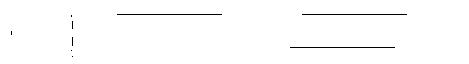


Similarly for treatments in prison:


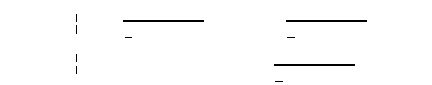


# Parameterising the incarceration component of the model

An adapted incarceration model without HCV transmission, ‘deaths’ and ‘births’ (model schematic in Supplementary Figure 1) was used to calibrate the incarceration component of the model to data from NESI on the proportion of community PWID with 0, 1 or multiple previous incarcerations. This adapted model stratified PWID by incarceration status (community or prison) and the number of previous incarcerations (0, 1 and 2 or more in the community; and 0 or 1 or more in prison). PWID are incarcerated and reincarcerated with rates
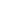
 and
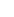
, respectively, which depend on whether PWID are recent (<5 years injecting) or non-recent (>5 years injecting) initiates of injecting. PWID are released from prison at a fixed rate
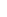
, with a proportion (
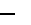
) of those being released leaving the model due to increased mortality post-release. Using this adapted model, 1000 PWID were followed throughout their injecting ‘career’ for 20 years from the onset of injecting, with a proportion
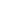
 of the 1000 PWID starting in each compartment. These proportions are then used to find the proportion of PWID that initiate injecting in each incarceration compartment in the full model.

Supplementary Figure 1: Schematic for the incarceration sub-model.

We used an Approximate Bayesian computation sequential Monte Carlo (ABC SMC) algorithm(6) to obtain a sample of incarceration-related parameter sets that fit the NESI incarceration data sufficiently well whilst also giving rise to a total PWID population size that is within the latest estimated range for Scotland(7) (more details below). Supplementary Figure 2 shows an example fit obtained using the ABC SMC method along with the data points used in this fitting scheme and their 95% confidence intervals(8).

**
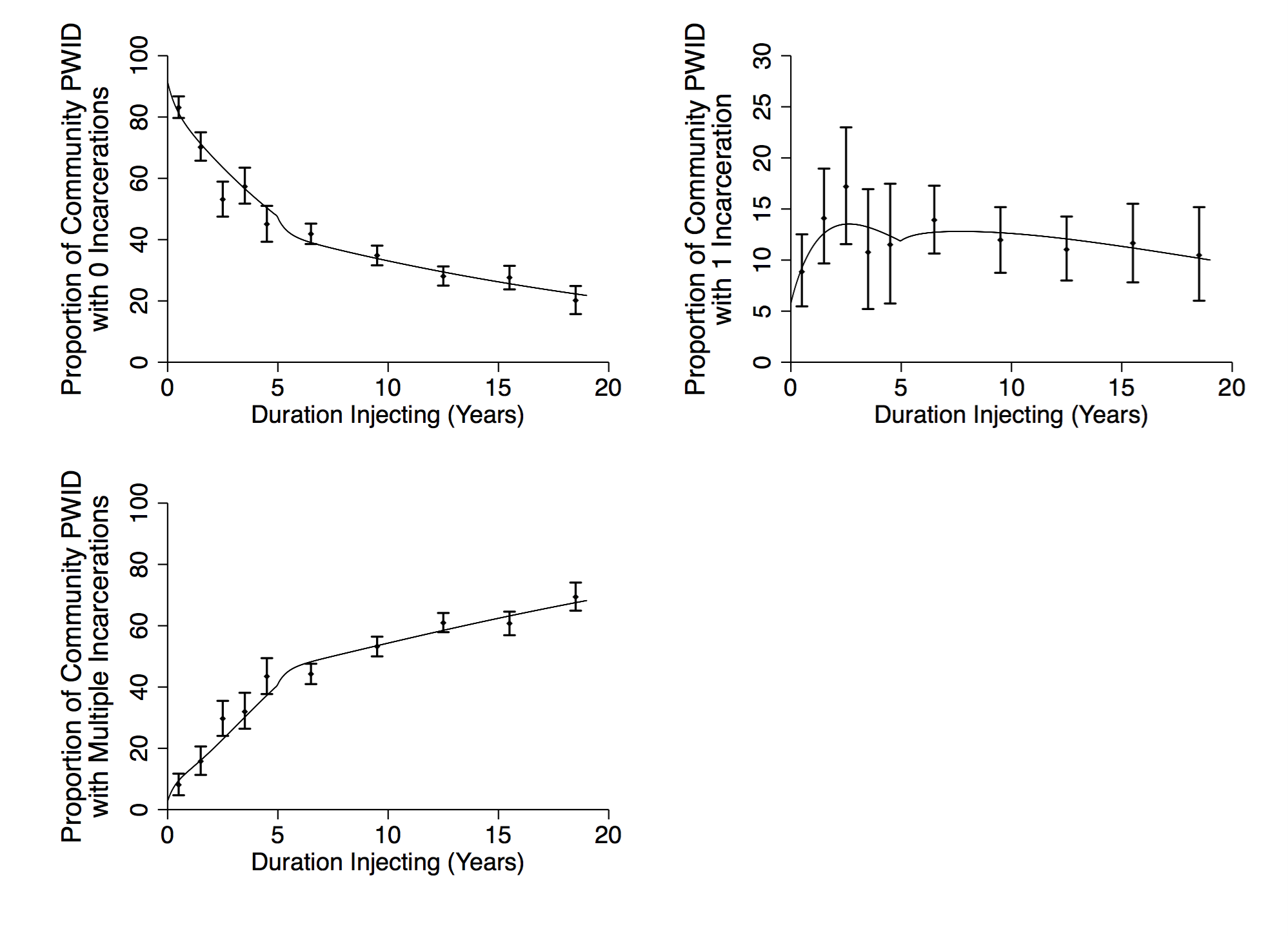
**

Supplementary Figure 2: Example of ABC SMC fit, along with the data points used in the fitting procedure with their 95% confidence intervals.

## The ABC SMC Algorithm

The aim of ABC methods is to infer posterior distributions where likelihood functions are difficult to compute/write down. In standard ABC methods, a set of parameters, θ^*^, is sampled from a prior distribution, π(θ), which is used in the model to generate a dataset x^*^ which is compared to experimental/observed data x_0_ using a distance function, d(x^*^,x_0_). If the distance between the generated data and observed data is less than a predetermined tolerance, ε, the sample, θ^*^, is accepted. The output is a sample from the distribution π(θ | d(x_0_,x^*^) ≤ ε), which if ε, is sufficiently small is a good approximation to the posterior distribution, π(θ|x_0_).

We utilized an ABC SMC algorithm to avoid possible issues with traditional ABC methods such as the ABC rejection sampler or the ABC MCMC algorithm, requiring less sampling to obtain the final population size and hence taking less time. In ABC SMC, a population of N sampled parameter sets, {θ^(1)^, θ^(2)^, …θ^(3)^}, sampled from the prior distributions, π(θ) are propagated through a sequence of intermediate distributions, π(θ | d(x_0_,x^*^) ≤ ε_i_), i=1 … T-1, until it represents a sample from the target distribution π(θ | d(x_0_,x^*^) ≤ ε_T_). The tolerances, ε_i_ are chosen so that ε_1_ > ε_2_ ... > ε_T_. The ABC SMC in its general form, as outlined in (6) proceeds as follows,

1. Initialize tolerances, ε_1_ ... ε_T_.

Set the population indicator t=0.

1. Set the particle indicator i=1.
2. If t=0 sample θ^**^ independently from π(θ).

Else, sample θ^*^ from the previous population {
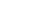
} with weights
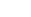
 and perturb the particle to obtain θ^** ~^ K_t_ (θ | θ^*^), where K_t_ is a perturbation kernel.

If π(θ^**^) = 0, return to STEP 3

Simulate a candidate dataset, x^*^, using the model.

If d(x_0_,x^*^) ≥ ε_t_, return to step 3.

1. Set
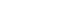
and calculate the weight for particle
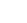
,


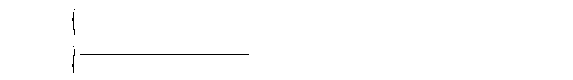


if i<N , set i=i+1, go to step 3.

1. Normalize the weights.

If t < T, set t=T+1, go to step 2.

## ABC SMC to sample incarceration parameters

In our implementation, we sought a sample of parameter sets that would approximately be from the posterior distribution π(θ |x_0_)
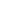
. The data, x_0_ (see Supplementary Table 1), consists of data points by {
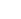
 which are the proportions of PWID in the community that have never been incarcerated (i=0), been incarcerated once (i=1), and been incarcerated multiple times (i=2) by duration injecting, t. We took the distance function to be the sum of the square errors on the logarithmic scale, i.e.
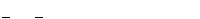
, where we denote our simulated data by
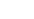
.

Supplementary Table 1: Data on the proportions of PWID with zero, one or multiple incarcerations by duration of injecting, used in the ABC SMC.

|  | Proportion of PWID who have | | |
| --- | --- | --- | --- |
| Duration of injecting (Years) | Never been incarcerated | Been incarcerated once | Been incarcerated multiple times |
| <=1 | 83.07 | 8.85 | 8.07 |
| >1 and <=2 | 70.17 | 14.09 | 15.75 |
| >2 and <=3 | 53.13 | 17.19 | 29.69 |
| >3 and <=4 | 57.29 | 10.76 | 31.94 |
| >4 and <=5 | 45.05 | 11.50 | 43.45 |
| >5 and <=8 | 41.85 | 13.92 | 44.24 |
| >8 and <=11 | 34.83 | 11.97 | 53.21 |
| >11 and <=14 | 28.04 | 11.03 | 60.93 |
| >14 and <=17 | 27.61 | 11.67 | 60.74 |
| >17 and <=20 | 20.16 | 10.47 | 69.37 |

In order to ensure parameter sets gave rise to expected population sizes (within a recently estimated range(7)), a sampled parameter set, θ^**^, was accepted to be part of population t, if the distance between the generated data and observed data was less than ε_t_, using the above distance function, and the model population size was within the range [11500, 18600]. For each parameter set, the model population size was found by calibrating the number of incarcerated PWID at equilibrium to that suggested by Scottish prison data; the proportion of Scottish prisoners who are current PWID (P – sampled as part of the ABC SMC regime) and the average number of people imprisoned at any one time in Scotland (7853 in 2010/11 (9)).

Supplementary Table 2: Prior distributions and perturbation kernels for the ABC SMC algorithm used in the uncertainty analysis.

| Parameter | | Symbol | Prior Distribution | Perturbation Kernel | Source & Comments |
| --- | --- | --- | --- | --- | --- |
| Death rate (per year) | | μ_1_ | Sampled from a Poisson distribution with mean (10), with sampled values divided by 1000. | Uniform  (-0.0005,0.0005) | (10) |
| Average duration Injecting (years) | | μ_2_ | Uniform on (5,20) | Uniform  (-0.375, 0.375) | (11) |
| Factor increase in mortality for 2 weeks following prison release | | μ* | Lognormal with parameters (2.0053,0.1393) truncated to 95% confidence interval [5.7,9.9] | Uniform  (-0.105,0.105) | (12) |
| Percentage of prison population that are current PWID | | P | Normal with parameters (0.19, .006) truncated to 95% CI (0.18 - 0.21) | Uniform  (-0.0012,0.0012) | Estimated from prison Survey |
| Proportion of PWID initiating injecting | |  | Dirichlet distribution with parameters (10,1,1,1,1) |  | Obtained through model fitting.  In the final model which does not stratify incarceration history into incarcerated once and twice or more:   - p_2_ and p_4_ are combined to give the proportion of PWID initiating in prison - p_3_ and p_5_ are combined to give the proportion of PWID initiating in the community having been incarcerated – a random proportion of which have been recently released. |
|  | Community, never incarcerated | p_1_ |  | Uniform  (-0.04 , 0.04) |
|  | Incarcerated for first time | p_2_ |  | Uniform  (-0.04 , 0.04) |
|  | Community, incarcerated once | p_3_ |  | Uniform  (-0.04 , 0.04) |
|  | Incarcerated for 2^nd^ or more time | p_4_ |  | Uniform  (-0.04 , 0.04) |
|  | Community, incarcerated twice or more | p_5_ |  | Uniform  (-0.04 , 0.04) |
| Incarceration rates per year | | γ |  |  | Obtained through model fitting |
|  | | <5 years injecting | Uniform on (0,0.25) | Uniform  (-0.025,0.025) |
|  | | >5 years injecting | Uniform on (0,0.25) | Uniform  (-0.025,0.025) |
| Re-incarceration rates per year | | δ |  |  | Obtained through model fitting |
|  | | <5 years injecting | Uniform on (0,1) | Uniform  (-0.05,0.05) |
|  | | >5 years injecting | Uniform on (0,1) | Uniform  (-0.05,0.05) |
| Release Rate | | τ | Normal with parameters (0.48, .019) truncated to 95% CI (0.44 - 0.52) | Uniform  (-0.0015 , 0.0015) | Estimated from prison survey. |

To ensure the gradual transition between populations, we set the tolerances to be equally spaced on the log-scale between 20 and 0.35 with 50 iterations of the algorithm (i.e. T=50), choosing ε_T_ to be 0.35 because a single best fit to the data gives an error (defined by the distance function) of 0.25. Prior distributions and perturbation kernels for each parameter sampled by the ABC SMC are shown in Supplementary Table 2.

# Parameterising the transmission component of the model

For each of the 10,000 parameter fits for the incarceration sub-model, parameter values for the proportion of infections that spontaneous clear, SVR rates for PEG-IFN/RBV treatments and the increased risk amongst recently released PWID were sampled from parameters distributions (Table 2). For each of these parameter sets, HCV incidences amongst recent and non-recent community PWID and incarcerated PWID were sampled from the distribution of incidence rates after removing those falling outside the 2.5 and 97.5 percentiles (section 3.2) and HCV transmission rates amongst recent and non-recent community PWID and incarcerated PWID were calibrated to these sampled values using a pattern-search algorithm (implemented in Matlab_R2015b). If these parameter sets projected HCV prevalence amongst community PWID and incarcerated PWID lay within the 95% confidence intervals of the corresponding data for NESI 2008 and the prison survey, they were accepted as model fits.

## Increased Risk amongst recently incarcerated PWID

Using data from 2 survey years (2008/9 and 2013) in the needle exchange surveillance initiative(13-15) (NESI) involving 1,717 anti-HCV negative PWID from across Scotland, a logistic regression was employed to investigate the association between recent incarceration (in the last 6 months) and recent HCV infection. Recent infection was defined as those who were tested anti-HCV negative and HCV-RNA positive on dried blood spot, whilst duration of injecting, categorized by less than or more than 5 years, was controlled for as a possible confounder. The central estimate of the generated adjusted odds ratio (i.e. 2.30; 95% CI 0.97-5.46) was taken to be the risk factor for recently incarcerated PWID in the baseline model, whilst the corresponding 95% confidence interval was utilized in the uncertainty analysis.

## HCV incidence amongst recent and non-recent PWID

Estimates for HCV incidence amongst current community and all incarcerated PWID along with 95% confidence intervals (Supplementary Table 3) were derived using the methods below, using data from NESI 2008(13) and the prison survey(16). Community current PWID were defined to be those that injected in the last 6 months. Incidence was estimated amongst all incarcerated PWID due to possible temporary cessation during incarceration. HCV incidence was derived using the formula,


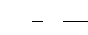


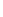
where T is the estimated duration of the window period (where the virus is detectable but prior to the formation of antibodies), n is the number of recent infections as defined above, and N is the number of anti-HCV negatives (susceptibles). A point estimate was derived using the mean duration of the window (51 days), while 95% confidence intervals were derived by a bootstrapping method in which:

1. 10000 values were sampled from a normal distribution relating to the window period (mean 51 days, variance 56 days), T, and a beta distribution (n, N-n) corresponding to the proportion of recent infections, p.
2. The above formula for incidence was used with each sampled pair from (i), using p in place of n/N, to generate a distribution of incidence rates.
3. Taking 2.5^th^ and 97.5^th^ percentile values from (ii) to generate upper and lower confidence limits.

Supplementary Table 3: Derived HCV incidence amongst recent and non-recent current community PWID and incarcerated PWID.

|  | Recent Infections (n) | Susceptibles  (N) | Derived Incidence (using 51 days as duration of window period) | Derived 95% Confidence interval |
| --- | --- | --- | --- | --- |
| Recent Community PWID (<5 years injecting): 2008 | 13 | 392 | 23.1 | 11.9-40.6 |
|  |  |  |  |  |
| Non-recent community PWID (>5 years injecting):2008 | 7 | 539 | 10.5 | 4.8-19.5 |
|  |  |  |  |  |
| Incarcerated PWID (2010/11) | 3 | 481 | 4.5 | 0.9-11.6 |

# Uncertainty in Model Projections

We undertook a linear regression analysis of covariance (ANCOVA) in order to determine which parameter uncertainties are influential in the model projections of the 15-year impact of scaling up annual prison treatment rates so that 80% of chronically infected PWID with at least 16-week sentences are treated on prison entry from 2015. The proportion of each model outcome’s sum-of-squares contributed by each parameter was calculated to estimate the importance of individual parameters to the overall uncertainty (Supplementary Table 4).

Supplementary Table 4: Contribution of parameters to uncertainty in model projections

| Parameter | | Symbol | Contribution to the total uncertainty in the model projections of the 15-year impact of scaling up annual prison treatment rates | |
| --- | --- | --- | --- | --- |
|  |  |  | On HCV incidence (%) | On chronic HCV prevalence (%) |
| Incarceration rates per year | | γ |  |  |
|  | Recent PWID (<5yrs injecting) |  | 3.119 | 3.121 |
|  | Non-recent PWID (>5yrs injecting) |  | 1.280 | 1.409 |
| Reincarceration rates per year | | δ |  |  |
|  | Recent PWID (<5yrs injecting) |  | 5.621 | 6.754 |
|  | Non-recent PWID (>5yrs injecting) |  | 7.503 | 7.660 |
| Percentage of PWID initiating injecting when | |  |  |  |
|  | Never incarcerated | p_1_ | 0.124 | 0.175 |
|  | Incarcerated | p_2_ | 0.124 | 0.175 |
|  | Recently released | p_3_ | 0.124 | 0.175 |
|  | Previously Incarcerated | p_4_ | 0.124 | 0.175 |
| Release rate from prison per year | | τ | 0.182 | 0.253 |
|  |  |  |  |  |
| Increased risk amongst recently released PWID (<6 months since release) | | η | 17.292 | 11.894 |
|  |  |  |  |  |
| Proportion of new infections that spontaneously clear | | α | 8.160 | 8.882 |
|  |  |  |  |  |
| Percentage of prison population that are current PWID | | P | 0.000 | 0.005 |
|  |  |  |  |  |
| PWID leaving rate (cessation+death) per year | | μ | 3.770 | 4.391 |
| Factor increase in mortality for 2 weeks following prison release | | μ* | 0.148 | 0.141 |
| HCV infection rate amongst | | λ |  |  |
|  | Recent Community PWID (<5yrs injecting) |  | 8.742 | 8.954 |
|  |  |  |  |  |
|  | Non-recent Community PWID (>5yrs injecting) |  | 24.442 | 25.166 |
|  |  |  |  |  |
|  | Incarcerated PWID |  | 1.303 | 0.196 |
|  |  |  |  |  |
| Percentage of incarcerated PWID with sentences >16 weeks | | ε | 17.944 | 20.474 |

**References**

1. Grebely J., Prins M., Hellard M., Cox A. L., Osburn W. O., Lauer G., . . . Hepatitis C. I. I. C. Hepatitis C virus clearance, reinfection, and persistence, with insights from studies of injecting drug users: towards a vaccine, The Lancet Infectious diseases 2012.

2. Martin N. K., Vickerman P., Foster G. R., Hutchinson S. J., Goldberg D. J., Hickman M. Can antiviral therapy for hepatitis C reduce the prevalence of HCV among injecting drug user populations? A modeling analysis of its prevention utility, Journal of hepatology 2011.

3. Vickerman P., Martin N., Turner K., Hickman M. Can needle and syringe programmes and opiate substitution therapy achieve substantial reductions in hepatitis C virus prevalence? Model projections for different epidemic settings, Addiction 2012.

4. Dore G. J., Feld J. J. Hepatitis C virus therapeutic development: in pursuit of "perfectovir", Clinical infectious diseases : an official publication of the Infectious Diseases Society of America 2015.

5. Aspinall E. J., Mitchell W., Schofield J., Cairns A., Lamond S., Bramley P., . . . Hutchinson S. J. A matched comparison study of hepatitis C treatment outcomes in the prison and community setting, and an analysis of the impact of prison release or transfer during therapy, Journal of viral hepatitis 2016.

6. Toni T., Welch D., Strelkowa N., Ipsen A., Stumpf M. P. Approximate Bayesian computation scheme for parameter inference and model selection in dynamical systems, J R Soc Interface 2009.

7. Overstall A. M., King R., Bird S. M., Hutchinson S. J., Hay G. Incomplete contingency tables with censored cells with application to estimating the number of people who inject drugs in Scotland, Stat Med 2014.

8. Glaz J., Sison C. P. Simultaneous confidence intervals for multinomial proportions, J Stat Plan Inference 1999.

9. Statistical bulletin - Crime and Justice series:Prison statistics Scotland: 2010-11 An National Statistics Publication for Scotland; 2011.

10. Hickman M., Hope V., Coleman B., Parry J., Telfer M., Twigger J., . . . Annett H. Assessing IDU prevalence and health consequences (HCV, overdose and drug-related mortality) in a primary care trust: implications for public health action, Journal of public health 2009.

11. Sweeting M., De Angelis D., Ades A., Hickman M. Estimating the prevalence of ex-injecting drug use in the population, Statistical methods in medical research 2009.

12. Merrall E. L., Kariminia A., Binswanger I. A., Hobbs M. S., Farrell M., Marsden J., . . . Bird S. M. Meta-analysis of drug-related deaths soon after release from prison, Addiction 2010.

13. Allen E. J., Palmateer N. E., Hutchinson S. J., Cameron S., Goldberg D. J., Taylor A. Association between harm reduction intervention uptake and recent hepatitis C infection among people who inject drugs attending sites that provide sterile injecting equipment in Scotland, The International journal on drug policy 2012.

14. Aspinall E., Hutchinson S. J., Taylor A., Palmateer N., Hellard M., Allen E., Goldberg D. Uptake of paraphernalia from injecting equipment provision services and its association with sharing of paraphernalia among injecting drug users in Scotland, Drug and alcohol dependence 2012.

15. Palmateer N. E., Taylor A., Goldberg D. J., Munro A., Aitken C., Shepherd S. J., . . . Hutchinson S. J. Rapid decline in HCV incidence among people who inject drugs associated with national scale-up in coverage of a combination of harm reduction interventions, PloS one 2014.

16. Taylor A., Munro A., Allen E., Dunleavy K., Cameron S., Miller L., Hickman M. Low incidence of hepatitis C virus among prisoners in Scotland, Addiction 2013.
